# Contralateral upper tract urothelial carcinoma after nephroureterectomy: the predictive role of DNA methylation

**DOI:** 10.1186/s13046-015-0120-2

**Published:** 2015-01-22

**Authors:** Lei Zhang, Gengyan Xiong, Dong Fang, Xuesong Li, Jin Liu, Weimin Ci, Wei Zhao, Nirmish Singla, Zhisong He, Liqun Zhou

**Affiliations:** Department of Urology, Peking University First Hospital, Institute of Urology, Peking University, National Urological Cancer Center, No. 8 Xishiku St, Xicheng District, Beijing, 100034 China; Beijing Institute of Genomics, Chinese Academy of Sciences, Beijing, 100101 China; Key Laboratory of Carcinogenesis and Translational Research (Ministry of Education), Department of Cell Biology, Peking University Cancer Hospital and Institute, Beijing, 100142 China; Department of Urology University of Texas Southwestern Medical Center, 5303 Harry Hines Blvd, Dallas, TX 75390 USA

**Keywords:** Methylation, Upper tract urothelial carcinoma, Contralateral recurrence, Radical nephroureterectomy, Predictors

## Abstract

**Background:**

Aberrant methylation of genes is one of the most common epigenetic modifications involved in the development of urothelial carcinoma. However, it is unknown the predictive role of methylation to contralateral new upper tract urothelial carcinoma (UTUC) after radical nephroureterectomy (RNU). We retrospectively investigated the predictive role of DNA methylation and other clinicopathological factors in the contralateral upper tract urothelial carcinoma (UTUC) recurrence after radical nephroureterectomy (RNU) in a large single-center cohort of patients.

**Methods:**

In a retrospective design, methylation of 10 genes was analyzed on tumor specimens belonging to 664 consecutive patients treated by RNU for primary UTUC. Median follow-up was 48 mo (range: 3–144 mo). Gene methylation was accessed by methylation-sensitive polymerase chain reaction, and we calculated the methylation index (MI), a reflection of the extent of methylation. The log-rank test and Cox regression were used to identify the predictor of contralateral UTUC recurrence.

**Results:**

Thirty (4.5%) patients developed a subsequent contralateral UTUC after a median follow-up time of 27.5 (range: 2–139) months. Promoter methylation for at least one gene promoter locus was present in 88.9% of UTUC. Fewer methylation and lower MI (P = 0.001) were seen in the tumors with contralateral UTUC recurrence than the tumors without contralateral recurrence. High MI (P = 0.007) was significantly correlated with poor cancer-specific survival. Multivariate analysis indicated that unmethylated RASSF1A (P = 0.039), lack of bladder recurrence prior to contralateral UTUC (P = 0.009), history of renal transplantation (P < 0.001), and preoperative renal insufficiency (P = 0.002) are independent risk factors for contralateral UTUC recurrence after RNU.

**Conclusions:**

Our data suggest a potential role of DNA methylation in predicting contralateral UTUC recurrence after RNU. Such information could help identify patients at high risk of new contralateral UTUC recurrence after RNU who need close surveillance during follow up.

## Background

Upper tract urothelial carcinoma (UTUC) accounts for about 5-10% of all urothelial carcinomas [1]. The standard treatment of upper tract urothelial carcinoma (UTUC) is radical nephroureterectomy (RNU) with bladder cuff excision [2]. Although the development of contralateral UTUC after RNU is relatively rare with an estimated incidence of 0.8-6% [3-12], the risk of consequent renal function compromise can be very grave, resulting in the need for dialysis. Few studies are available concerning the risk factors for predicting the development of metachronous contralateral UTUC [3-7]. These studies are limited by small sample sizes or unclear enrollment criteria, and only focusing on clinicopathological factors, it is conceivable that even tumors with identical clinicopathological characteristics could behave differently. A growing body of evidence indicates that aberrant methylation of cytosine-guanine dinucleotide (CpG) islands in the DNA promoter regions is one of the most common epigenetic modifications involved in the development of urothelial carcinoma [13-17]. Detecting gene promoter methylation may be a promising method for predicting contralateral UTUC recurrence after surgery.

Our previous study has demonstrated that renal transplant history and renal insufficiency are independent risk factors [3]. We updated the database and evaluated the methylation status of specific genes in UTUC in order to elucidate their value in predicting contralateral UTUC recurrence. Previous studies confirmed that TMEFF2, VIM and GDF15 were proved highly methylated level in UTUC, but the predictive role of them were not completely understood for the small sample size [17]. THBS1 is a known angiogenesis inhibitor associated with neovascularization in human cancer [18], its expression associated with tumor malignance in UTUC [19]. Because bladder urothelial carcinoma and UTUC both derive from the urothelial cell and show genomic and clinical similarities [20]. We chosen another six epigenetic biomarkers that accurately detect bladder cancer, including SALL3, ABCC6, RASSF1A, BRCA1, CDH1 and HSPA2 [15,16]. Finally, we selected the above 10 genomic regions wih CpG islands to investigate the predictive role in new contralateral UTUC after RNU. To our knowledge, we are the first group to investigate the correlation between metachronous contralateral UTUC and gene methylation.

## Methods

### Patient selection

We retrospectively reviewed the records of 820 consecutive patients from Peking University First Hospital (Beijing, China) who underwent surgery for UTUC from 1999 to 2011. Of this cohort, 156 patients were excluded from the study: 76 for concomitant or previous bladder tumor, 30 for bilateral UTUCs, 23 for undergoing nephron-sparing surgery instead of RNU, and 27 for inability to extract DNA as there was only one paraffin specimen stored in our bank. 664 patients were ultimately included for evaluation. No patients with solitary kidney or vesicoureteral reflux were discovered in this cohort. No patients received neoadjuvant chemotherapy or prophylactic postoperative intravesical instillation chemotherapy, although adjuvant chemotherapy or radiotherapy was administered to some patients when evidence of metastasis or retroperitoneal recurrence was discovered.

### Patient evaluation

In this cohort, UTUC was diagnosed by computed tomography (CT), magnetic resonance imaging (MRI), urologic ultrasound, or ureteroscopy with or without biopsy. The contralateral upper tract was evaluated to exclude bilateral carcinoma involvement. Ipsilateral hydronephrosis was determined by ultrasound, MRI, or CT before operation. Based on the site of the dominant lesion, tumor location was divided into renal pelvic tumors and ureteral tumors, where ureteral tumors were further subdivided into the upper ureter (superior to the sacrum), the middle ureter (between the upper and the lower borders of the sacrum), and the lower ureter (inferior to the sacrum) [21]. The preoperative estimated glomerular filtration rate (eGFR) was evaluated by the modified estimating equation of glomerular filtration rate for Chinese patients [eGFR (ml/min/1.73 m2) = 175 × Scr^− 1.234^ × age^− 0.179^ (×0.79 for female)] [22].

All surgical specimens were reviewed by two senior pathologists who were blinded to the patients’ personal data. Tumor stage was classified based on the 2002 UICC TNM classification of malignant tumors. Histological grades were assessed based on the WHO classification of 1973. Tumor architecture was divided into papillary and sessile groups. Tumor multifocality was described as the simultaneous existence of two or more pathologically validated macroscopic tumors from any location.

### Methylation analyses of gene promoters

Eight 5-um-thick sections were obtained from formalin-fixed paraffin-embedded tumor tissue, DNA was extracted from them containing >70% tumor using the QIAamp DNA FFPE Tissue Kit (Qiagen, Hilden, Germany). For bisulfite transformation, approximately 1.5 μg tumor DNA was treated with sodium bisulfite using EpiTect Fast Bisulfite Conversion Kits (Qiagen, Hilden, Germany). Sample processing was performed according to the manufacturer’s protocol. Methylation-sensitive polymerase chain reaction was performed using approximate 50 ng of converted DNA to analyze the methylation status of the 10 gene promoters, as previously described by Herman et al. [23]. We performed PCR for methylated and also unmethylated sequences. The PCR conditions and primers used for each gene is shown in Table 1. We used commercially available methylated human genomic DNA (Qiagen, Hilden, Germany) as positive control, and we used water blanks and PCR mixtures as negative control.

**Table 1:** **Primer sequences for MSP**

**Gene**	**Sense 5'→3'**	**Antisense 5'→3’**	**Annealing temperature (°C)**	**Product size (bp)**
ABCC6 M	GGCGTTCGGGGAGTT	CGACCTCGACCCGATAAT	57	247
ABCC6 U	AGGTGTTTGGGGAGTTGG	TCTCAACCTCAACCCAATAATC	58	244
BRCA1 M	TCGTGGTAACGGAAAAGCGC	AAATCTCAACGAACTCACGCC	58	75
BRCA1 U	TTGGTTTTTGTGGTAATGGAAAAGTGT	CAAAAAATCTCAACAAACTCACACCA	56	86
CDH1 M	GTGGGCGGGTCGTTAGTTTC	CTCACAAATACTTTACAATTCCGACG	57	172
CDH1 U	GGTGGGTGGGTTGTTAGTTTTGT	AACTCACAAATCTTTACAATTCCAAC	59	172
GDF15 M	CGGCGGTTATTTGTATTTGC	AACGATCGTATCACGTCCC	60	132
GDF15 U	ATTTGGTGGTTATTTGTATTTGT	AACAATCATATCACATCCCACA	57	135
HSPA2 M	TAAGAATCGGGAATTGGGC	AATCGATACCGATAACCGAA	58	172
HSPA2 U	TTATAAGAATTGGGAATTGGGT	AAATCAATACCAATAACCAAA	55	176
RASSF1A M	GGGTTTTGCGAGAGCGCG	GCTAACAAACGCGAACCG	64	169
RASSF1A U	GGTTTTGTGAGAGTGTGTTTAG	CACTAACAAACACAAACCAAAC	59	169
SALL3 M	GTTCGCGTAGTCGTCGTC	TACTCGAAAACCCCGTCA	57	203
SALL3 U	GTGGTTTGTGTAGTTGTTGTTGTT	CCCAACCCTCACCATACTC	57	220
THBS1 M	TGCGAGCGTTTTTTTAAATGC	TAAACTCGCAAACCAACTCG	62	74
THBS1 U	GTTTGGTTGTTGTTTATTGGTTG	CCTAAACTCACAAACCAACTCA	62	115
TMEFF2 M	GAAGAGGGGCGTTAGTTC	ACGCTAACCCGAATAAAACT	57	151
TMEFF2 U	GGAAGAGGGGTGTTAGTT	AACACTAACCCAAATAAAACT	55	153
VIM M	TTATAAAAATAGCGTTTTCGGC	ATAACGCGAACTAACTCCCG	59	143
VIM U	GGGTTATAAAAATAGTGTTTTTGGT	ACAATAACACAAACTAACTCCCA	56	149

Because a low prevalence of hypermethylation for all these gene promoters was validated in normal tissue [13-16,24], we did not compare the methylation status between UTUC and normal tissue any further in our study. As previous described [14], the extent of methylation for each tumor was calculated by a methylation index (MI; the ratio of number of methylated genes/total number of analyzed genes).

### Postoperative follow-up

Postoperative follow-up consisted of history, physical examination, urinalysis, serum creatinine, urine cytology (and/or urine fluorescence in situ hybridization), chest x-ray, cystoscopy, and ultrasound or CT/MRI. The follow-up interval was every 3 months for the first 2 years and yearly thereafter at our institute.

Contralateral UTUC after RNU was defined as urothelial carcinoma in the contralateral upper urinary tract detected on imaging or confirmed by pathological evaluation. The endpoint of this study was the first detection of contralateral UTUC or, for patients who did not develop such an outcome, the date of last follow-up or death.

### Statistical analysis

Categorical data was analyzed using Pearson’s chi-squared test. Continuous data were analyzed using the Mann–Whitney U test and Kruskal-Wallis H test. Log-rank tests were used for univariate analysis, and Cox’s proportional hazards regression model was used for multivariate analysis. Only variables identified as significant by univariate analysis were analyzed in a multivariate fashion. Statistical analysis was performed using SPSS 20.0 (IBM, Corp, Armonk, NY, USA). *P* value <0.05 was regarded as significant.

## Results

### Overall results of Clinical follow-up

All 664 patients included were proven to have UTUC pathologically. The patients’ demographic and histological data are presented in Table 2. The median age of our cohort was 68 years (range 20–90). The median follow-up period was 48 months (range 3–144). Thirty (4.5%) patients developed a subsequent contralateral UTUC after a median follow-up time of 27.5 (range: 2–139) months. The 5-year probability of freedom from contralateral metachronous UTUC was 95%. The mean contralateral UTUC-free survival period was 136 ± 1.488 months. 223 patients (33.6%) developed bladder recurrence. 214 patients (32.2%) died of urothelial cancer after a median follow-up time of 32 (range 2–143) months; the median survival period was 115 months (95% CI, 99–130 months).

**Table 2:** **Patient demographic and histological data**

**Variables**	**N (%)**
Gender	
Male	295 (44.4)
Female	369 (55.6)
Age	
<70	372 (56.0)
≥70	292 (44.0)
Preoperative renal function	
eGFR ≥ 60	298 (44.9)
60 >eGFR ≥15	316 (47.6)
eGFR <15	50 (7.5)
Side	
Left	326 (49.1)
Right	338 (50.9)
Transplant recipient	
No	653 (98.3)
Yes	11 (1.7)
Ipsilateral hydronephrosis	
Absence	290 (43.7)
Presence	374 (56.3)
Tobacco consumption	
No	543 (81.8)
Yes	121 (18.2)
Surgical approach	
Open	445 (67.0)
Laparoscopic	219 (33.0)
Tumor size	
≤3 cm	373 (56.2)
>3 cm	291 (43.8)
Architecture	
Papillary	515 (77.6)
Sessile	149 (22.4)
Ureteroscopy	
No	583 (87.8)
Yes	81 (12.2)
Location	
Pelvis	368 (55.4)
Ureter	296 (44.6)
Upper ureter	58 (8.7)
Middle ureter	68 (10.2)
Lower ureter	170 (25.6)
Multifocality	
No	505 (76.1)
Yes	159 (23.9)
CIS	
Absence	645 (97.1)
Presence	19 (2.9)
Contralateral recurrence	
No	634 (95.5)
Yes	30 (4.5)
Bladder recurrence	
No	441 (66.4)
Yes	223 (33.6)
Before contralateral UTUC	209 (31.5)
Concomitant and after contralateral UTUC	14 (2.1)
Tumor stage	
Ta or T1	221 (33.3)
T2	237 (35.7)
T3	194 (29.2)
T4	12 (1.8)
Tumor grade	
G1	21 (3.2)
G2	360 (54.2)
G3	283 (42.6)
N status	
N+	47 (7.1)
cN0 or pN0	617 (92.9)
Adjuvant therapy	
No	641 (96.5)
Yes	23 (3.5)

eGFR = estimated glomerular filtration rate; CIS = carcinoma in situ; UTUC = upper tract urothelial carcinoma.

### CpG island Methylation for UTUC

Aberrant methylation for at least one gene promoter locus was detected in 88.9% of all DNA samples. For all loci, more frequent methylation was present in patients without contralateral UTUC recurrence than patients with contralateral recurrence with statistical significance achieved for RASSF1A (*P* = 0.012) and VIM (*P* = 0.006). The methylation rate of RASSF1A is 27.4% (174/634 cases) in patients without contralateral UTUC recurrence, and only 6.7% (2/30 cases) in patients with contralateral UTUC recurrence. For VIM, the methylation rate is 64.7% (410/634) in patients without contralateral UTUC recurrence, and 40% (12/30 cases) in patients with contralateral UTUC recurrence. The frequency of methylation for each gene and the correlation between methylation and patients characteristics were shown in Table 3. The methylation of VIM (P = 0.008), BRCA1 (P = 0.005), TMEFF2 (P = 0.003), and RASSF1A (P = 0.037) was correlated with the age, gender, tumors size, and mulifocality, respectively. Higher methylation rate was present in nonsmoker than smokers for BRCA1 (P = 0.019) and SALL3 (P = 0.036). The sessile architecture preponderance of methylation was seen in ABCC6 (P = 0.025), BRCA1 (P = 0.000), CDH1 (P = 0.001), HSPA2 (P = 0.014), RASSF1A (P = 0.045), and TSHB1 (P = 0.000), while higher GDF15 (P = 0.002) methylation rate was seen in UTUC with papillary architecture. Hypermethylation was associated with advanced tumor grade for BRCA1 (P = 0.003), CDH1 (P = 0.002), HSPA2 (P = 0.009), RASSF1A (P = 0.017), and THBS1 (P = 0.000), while methylated GDF15 (P = 0.002) promoter was associated with low tumor grade. Hypermethylation was associated with advanced tumor stage for ABCC6 (P = 0.050), BRCA1 (P = 0.032), CDH1 (P = 0.004), GDF15 (P = 0.005), HSPA2 (P = 0.000), RASSF1A (P = 0.003), TSHB1 (P = 0.018), and TMEFF2 (P = 0.002).

**Table 3:** **Summary of the frequency of methylation of the genes in UTUC based on the main clinicopathologic variables**

	**Overall methylation (%) n = 664**	**Age, %**		**Gender, %**		**Architecture, %**		**Tumor size, %**		**Multifocal, %**		**Smoke, %**		**Stage** ^**a**^ **, %**		**Grade** ^**b**^ **, %**	
		**<70 n = 372**	**≥70 n = 292**	**Male n = 295**	**Female n = 369**	**Papillary n = 515**	**Sessile n = 149**	**≤3 cm n = 373**	**>3 cm n = 291**	**Yes n = 159**	**No n = 505**	**Yes n = 121**	**No n = 543**	**Low stage n = 458**	**High stage n = 206**	**Low grade n = 381**	**High stage n = 283**
ABCC6	14.5	12.6	16.8	11.5	16.8	**12.8**	**20.1**	13.7	15.5	11.3	15.4	9.9	15.5	**12.7**	**18.4**	12.6	17.0
BRCA1	17.2	17.2	17.1	**12.5**	**20.9**	**14.0**	**28.2**	16.4	18.2	20.1	16.2	**9.9**	**18.8**	**15.1**	**21.8**	**13.4**	**22.3**
CDH1	14.2	13.2	15.4	14.2	14.1	**11.7**	**22.8**	14.5	13.7	11.9	14.9	15.7	13.8	**11.6**	**19.9**	**10.5**	**19.1**
GDF15	49.2	48.4	50.3	53.2	46.1	**52.4**	**38.3**	46.4	52.9	49.7	49.1	55.4	47.9	**45.6**	**57.3**	**54.3**	**42.4**
HSPA2	41.6	38.4	45.5	40.0	42.8	**39.0**	**50.3**	39.9	43.6	38.4	42.6	37.2	42.5	**35.2**	**55.8**	**37.3**	**47.3**
RASSF1A	26.5	26.1	27.1	25.4	27.4	**27.4**	**32.9**	24.1	29.6	**20.1**	**28.5**	19.8	27.8	**22.9**	**34.0**	**22.8**	**31.1**
SALL3	34.6	33.3	36.3	33.2	35.8	33.4	39.6	35.1	34.0	30.8	35.8	**26.4**	**36.5**	32.8	38.8	33.3	36.4
THBS1	25.2	22.8	28.1	26.4	24.1	**20.7**	**40.3**	27.6	22.0	21.4	26.3	26.6	24.9	**22.5**	**31.1**	**19.2**	**33.2**
TMEFF2	43.4	41.1	46.2	43.4	43.4	41.7	49.0	**38.3**	**49.8**	37.7	45.1	41.3	43.8	**39.3**	**52.4**	40.4	47.3
VIM	63.6	**59.1**	**69.2**	66.4	61.2	63.5	63.8	60.6	67.4	61.6	64.2	66.1	63.0	61.4	68.4	64.0	62.9

^a^The stage was divided into a low stage (stage Ta, T1, or T2) group and a high stage (stage T3 or T4) group.

^b^The grade was divided into a low grade (grade G1 or G2) group and a high grade (grade G3) group.

The data reaching for statistical significance was present in bold type.

The methylation of VIM (*P* = 0.008), BRCA1 (*P* = 0.005), TMEFF2 (*P* = 0.003), and RASSF1A (*P* = 0.037) was correlated with the age, gender, tumors size, and mulifocality, respectively. The methylation of BRCA1 (*P* = 0.019) and SALL3 (*P* = 0.036) were correlated with smoke history. The methylation of ABCC6 (*P* = 0.025), BRCA1 (*P* = 0.000), CDH1 (*P* = 0.001), GDF15 (*P* = 0.002), HSPA2 (*P* = 0.014), RASSF1A (*P* = 0.045), and TSHB1 (*P* = 0.000) was correlated with the architecture of tumors. Methylation was associated with advanced tumor grade for BRCA1 (*P* = 0.003), CDH1 (*P* = 0.002), HSPA2 (*P* = 0.009), RASSF1A (*P* = 0.017), and THBS1 (*P* = 0.000), and unmethylated GDF15 (*P* = 0.002) promoter was associated with advanced tumor grade. Methylation was associated with advanced tumor stage for ABCC6 (*P* = 0.050), BRCA1 (*P* = 0.032), CDH1 (*P* = 0.004), GDF15 (*P* = 0.005), HSPA2 (*P* = 0.000), RASSF1A (*P* = 0.003), TSHB1 (*P* = 0.018), and TMEFF2 (*P* = 0.002).

### MI for UTUC

**Figure 1:**
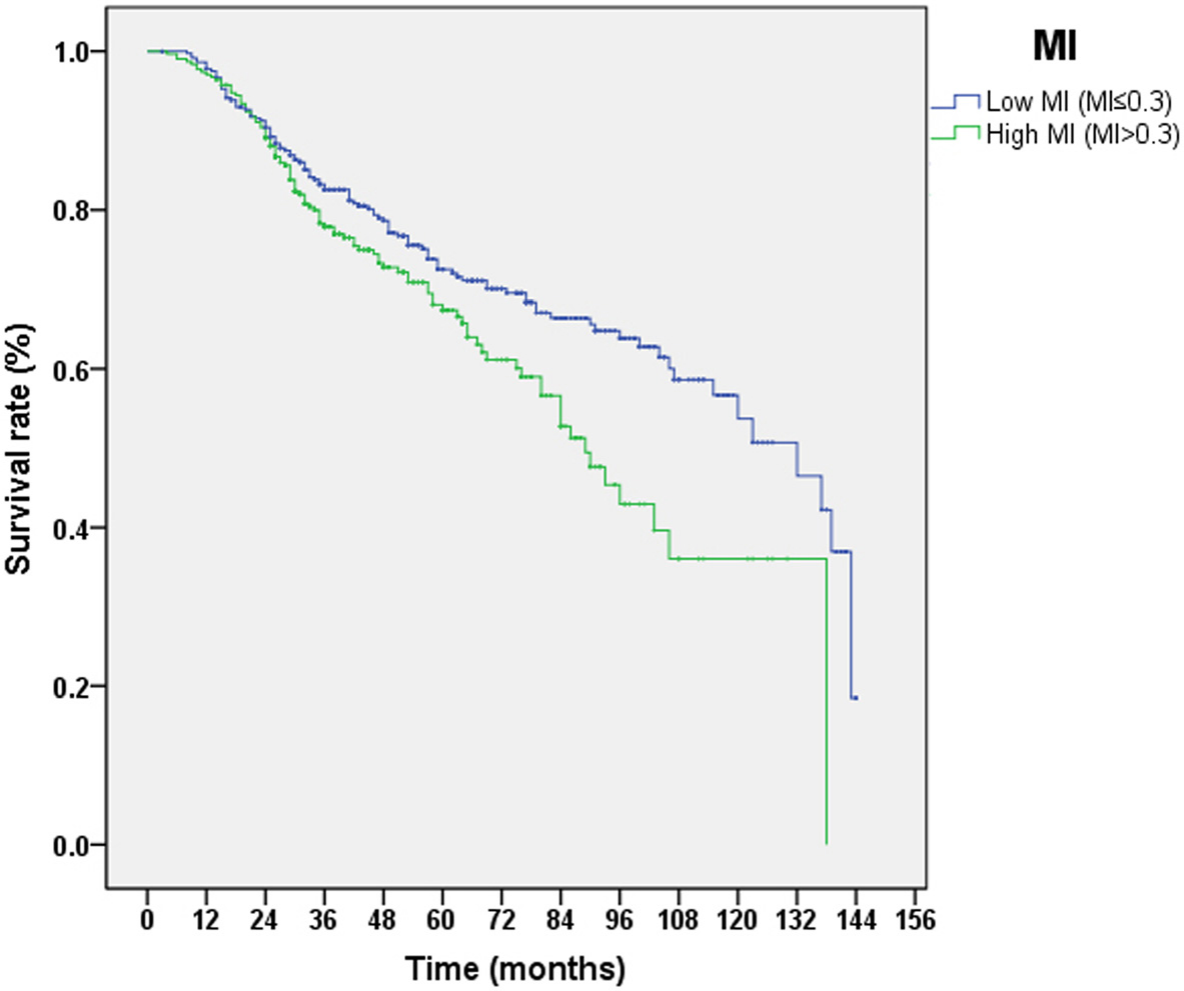
**Cancer-specific survival curves for MI.**

The MI based on the specific clinicopathological factor is presented in Table 4. Higher MI was seen in tumors with poorer prognostic factors, such as advanced grade (P < 0.001), increased stage (*P <* 0.001), positive lymph nodes (*P* = 0.014) or sessile architecture (*P* = 0.001). Lower MI was present in patients with hydronephrosis (*P* = 0.015), tumor located in lower ureter (*P <* 0.001), or those with history of renal transplantation (*P* = 0.011). The MI of tumors with contralateral UTUC recurrence was lower than for tumors without contralateral recurrence (*P* = 0.001). The median MI value was 0.3, and the cohort was divided into a low-MI group (MI value ≤0.3) and a high-MI group (MI value >0.3). Patients in the high-MI group had a poor cancer-specific survival (CSS, *P* = 0.007, Figure 1) than patients in the low-MI group.

**Table 4:** **MI based on the clinicopathological factors**

	**No. of patients**	**MI**		**P**
		**Mean**	**SD**	
Contralateral recurrence				0.001*
No	634	0.3361	0.22882	
Yes	30	0.1967	0.19025	
Grade				<0.001*
G1	21	0.1667	0.14606	
G2	360	0.3161	0.22121	
G3	283	0.3594	0.23732	
Stage				<0.001*
Ta or T1	221	0.3009	0.21062	
T2	237	0.2975	0.21803	
T3	194	0.3964	0.24629	
T4	12	0.4250	0.24541	
N status				0.014*
cN0 or pNo	617	0.3241	0.22860	
N+	47	0.4043	0.22259	
Architecture				0.001*
Papillary	515	0.3140	0.22534	
Sessile	149	0.3846	0.23358	
Ipsilateral hydronephrosis				0.015*
Absence	290	0.3545	0.23248	
Presence	374	0.3107	0.22461	
Main tumor location				0.000*
Pelvic	368	0.3677	0.23324	
Upper ureter	58	0.2914	0.22027	
Middle ureter	68	0.2824	0.21644	
Lower ureter	170	0.2800	0.21334	
Transplant recipient				0.011*
No	653	0.3326	0.22861	
Yes	11	0.1636	0.19117	

MI = methylation index.

*Statistically significant.

### Predictive factors of contralateral UTUC recurrence

**Figure 2:**
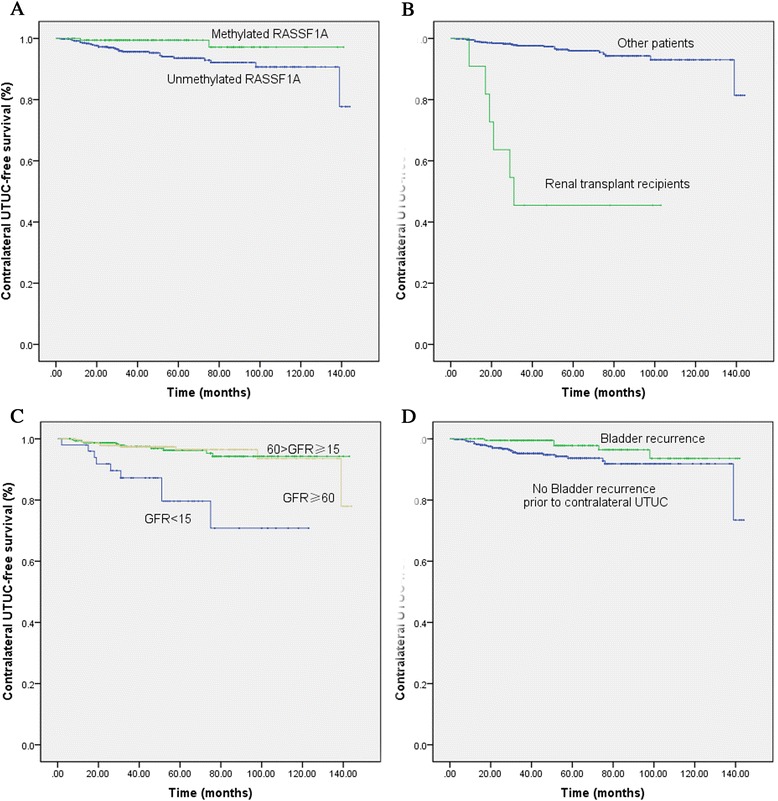
**Estimated contralateral UTUC-free Kaplan-Meier survival curves stratified by methylation status of RASSF1A (A,** ***P*** **= 0.019), renal transplant history (B,** ***P <*** **0.001), preoperative renal function (C,** ***P <*** **0.001), bladder recurrence (D,** ***P*** **= 0.032).**

Univariate analysis and multivariate analysis for contralateral UTUC recurrence after RNU is summarized in Tables 5 and 6, respectively. In multivariate analysis, unmethylated RASSF1A (*P* = 0.039, Figure 2A), renal transplant history (*P <* 0.001, Figure 2B), preoperative renal insufficiency (*P* = 0.002, Figure 2C), and lack of bladder recurrence before the development of contralateral UTUC (*P* = 0.007, Figure 2D) were found to be independent risk factors of contralateral UTUC recurrence after RNU.

**Table 5:** **Univariate analysis for contralateral UTUC recurrence after RNU**

**Variable**	**Univariate**	
	**Chi-square**	**P** ^**a**^
Gender	2.051	0.152
Age	2.256	0.112
Renal function	27.746	<0.001*
Side	0.140	0.708
Transplant recipient	74.902	<0.001*
Ipsilateral hydronephrosis	1.367	0.242
Smoke	0.063	0.802
Surgical approach	1.891	0.169
Tumor size	0.817	0.366
Architecture	0.737	0.391
Ureteroscopy	0.828	0.363
Location	2.015	0.569
Mulifocality	0.536	0.464
CIS	0.899	0.343
Bladder recurrence^b^	4.612	0.032*
Tumor stage	1.104	0.776
Tumor grade	1.730	0.421
N status	0.230	0.631
Adjuvant therapy	0.015	0.902
ABCC6	1.963	0.161
BRCA1	0.226	0.634
CDH1	2.226	0.136
GDF15	1.077	0.299
HSPA2	0.084	0.772
RASSF1A	5.481	0.019*
SALL3	1.696	0.193
THBS1	2.971	0.085
TMEFF2	1.072	0.301
VIM	5.004	0.025*
MI^c^	3.918	0.048*

^a^Kaplan Meier test was used for univariate analysis.

^b^Only bladder recurrence before contralateral UTUC recurrence was analyzed.

^c^ Because the MI correlated with methylation status of RASSF1A (r = 0.412, *P* = 0.000) and VIM (r = 0.478, *P* = 0.000), the MI was not analyzed in the multivariate models.

*Statistically significant.

**Table 6:** **Multivariate analysis for contralateral UTUC recurrence after RNU**

**Variable**	**Multivariate**		
	**HR**	**95% CI**	**P**
Renal function	0.418	0.242-0.723	0.002*
Transplant recipient	20.914	8.208-53.284	<0.001*
Bladder recurrence^a^	0.258	0.097-0.691	0.007*
RASSF1A	0.218	0.051-0.927	0.039*
VIM			0.278

*Statistically significant.

^a^Only bladder recurrence before contralateral UTUC recurrence was analyzed.

### Clinical characteristics of patients with contralateral UTUC recurrence

Six patients were renal transplant recipients, and 25 patients had no bladder recurrence before developing contralateral UTUC recurrence. Twenty-six patients were treated surgically for the contralateral UTUC at our institute, 14 with contralateral RNU and the other 12 with nephron-sparing treatment, including partial ureterectomy in 7 cases and endoscopic ablation in 5 cases. The other 4 patients with contralateral UTUC recurrence were not treated surgically because of poor physical status. Thirteen patients were confirmed to have taken Chinese herbs containing aristolochic acid for at least 6 months, 2 patients denied consuming any herbs, and the other patients could not provide detailed information of the herbs that they had taken. By the end of follow-up, there were 16 deaths in patients with contralateral UTUC recurrence, and 13 patients died of urothelial cancer. The median cancer-specific survival time after contralateral UTUC recurrence was 60 months (95% CI 39–80).

## Discussion

In the present study, we report a recurrence rate of contralateral UTUC after nephroureterectomy of 4.5%, which is similar to previous studies [3-12]. To our knowledge, this is the first study to investigate the predictive role of aberrant methylation to predict contralateral UTUC after RNU. Unmethylated RASSF1A, no bladder recurrence before the development of contralateral UTUC, renal transplant history, and preoperative renal insufficiency were independent risk factors for contralateral UTUC recurrence after RNU.

The multifocality and recurrence of urothelial cancer has been explained by two hypothesis. One is the field cancerization hypothesis [25], in which independent multiclonal tumor develops due to carcinogenic exposure of entire urothelium. The alternative intraluminal seeding hypothesis [26] indicates that intraluminal implantation deriving from an initial clone of tumor cells is the mechanism of multifocality or recurrence. The field cancerization hypothesis theory may have a more important effect on the development of contralateral UTUC in this cohort for a few reasons. First, none of our patients suffered from vesicoureteral reflux; although some patients underwent ureteroscopy, no correlations have been shown between ureteroscopy and contralateral UTUC recurrence. Second, Chinese herbs containing aristolochic acid are used nationwide, which could bring nephrotoxic and carcinogenic toxins to induce neoplasms of the entire urothelial field [27]. Third, patients with bladder recurrence demonstrated no trend of developing contralateral UTUC.

RASSF1A, a member of RAS family of genes, is a putative tumor suppressor gene. Consistent with our results, hypermethylation of RASSF1A is generally considered to correlate with poor prognostic parameters, such as advanced tumor grade or stage [13,14,28-31], muscle invasion [14,31], and disease progression [13,28] in transitional cell carcinoma. However, we also found that unmethylated RASSF1A was an independent risk factor of contralateral UTUC recurrence. In consistent with our result, Casadio et al. [32] also found lower methylation in recurring versus non-recurring patients in non muscle invasive bladder cancer, with statistical significance for RASSF1A. This concordance suggests a relationship between lower methylation of RASSF1A and recurrence of urothelial cancer. For our results, we propose two possibilities to explain this phenomenon. First, as hypermethylation of RASSF1A was correlated with poor prognostic parameters, patients with RASSF1A hypermethylation may die from tumor dissemination before developing contralateral UTUC recurrence. Second, aristolochic acid was proven to be associated with UTUC [27]. In the current study, among all 30 patients with contralateral UTUC recurrence, at least 13 patients have taken Chinese herbs containing aristolochic acid for more than 6 months. Combining these results, we believe aristolochic acid-related UTUCs have the trend of developing the contralateral UTUC recurrence in this cohort. A previous study of UTUC in Balkan endemic nephropathy patients, which is believed to arise secondary to aristolochic acid use [33], revealed that a lower tumor stage (pTa-pT1) and grade predominated among UTUC patients from the endemic and adjacent but not the control settlements from 1957–1986 [34]. This result suggests that the aristolochic acid-related UTUCs may have lower malignancy potential. Our results revealed unmethylated RASSF1A was more prevalent in UTUCs with lower malignancy potential. Unmethylated RASSF1A may correlated with aristolochic acid-related UTUCs. Hence, unmethylated RASSF1A seemed to correlate with the development of contralateral UTUC recurrence. The role of RASSF1A methylation in the recurrence of urothelial cancer, and the relationship between RASSF1A and aristolochic acid related urothelial cancer require further research.

Consistent with previous study [13,14], we found the similarity that higher MI were more prevalent in tumors with poor prognostic parameter. Higher MI was also found to be correlated with poor CSS. Lower MI (P = 0.001) were seen in the tumors with contralateral UTUC recurrence than the tumors without contralateral recurrence. More cancer-specific death before the development of contralateral UTUC could explain the result. In addition, aristolochic acid-related UTUCs may have lower MI due to the characteristics of lower malignancy potential, which could be another potential reason for contralateral UTUC recurrence with lower MI as aristolochic acid was extremely likely involved in the development of contralateral UTUC in this cohort.

Lack of bladder recurrence before the contralateral UTUC recurrence was associated with the development of contralateral UTUC. Two previous studies presented that the history of bladder tumor was associated with the development of contralateral UTUC after surgery [5,6]. However, the patients with bladder tumors prior to the first UTUC were not excluded in these studies. Although both derive from the urothelial cell, UTUC and bladder cancer exhibit biological and behavioral differences [35]. In the current study, we exclude patients with concomitant or previous bladder tumors before RNU. Although data are limited, of the 223 patients developing bladder recurrence, at least 156 patients (70.0%) received intravesical instillation with epirubicin or pirarubicin, which may have reduced the incidence of contralateral UTUC recurrence.

Consistent with our previous study [3], renal transplant recipient history and preoperative renal insufficiency correlated with contralateral UTUC recurrence even after expanding the sample size and introducing gene promoter methylation status into analysis. Hence our research continues to provide strong evidence that for patients with renal transplant or with renal insufficiency who would likely require dialysis, prophylactic RNU is a reasonable choice after first RNU for unilateral UTUC.

There are several strengths to our study. To our knowledge, we are the first group to investigate the correlation between gene methylation and contralateral UTUC recurrence after RNU. Our results could conceivably help tailor surveillance and adjuvant treatment. Second, we attempted to homogenize our cohort and thereby decrease treatment bias by applying strict inclusion criteria and minimizing differences in inter-operator surgical technique. Third, we provide a support that intravesical instillation with epirubicin or pirarubicin after RNU may reduce the incidence of contralateral UTUC recurrence. Finally, we once again confirmed strong evidence supporting prophylactic contralateral nephroureterectomy for end-stage renal failure and renal transplant recipient patients.

Our study nonetheless exhibits several limitations. Our study is limited by the inherent limitations of a retrospective approach, including selection bias and incomplete information of some valuable variables such as the consumption of Chinese herbs for all patients. Additionally, the relative short follow-up duration may not reveal accurate outcomes on survival analysis. The lack of relevant gene methylation function analysis further reduces the strength of this study. Additional studies including gene methylation function analysis and investigations on the correlation between methylation and aristolochic acid-related UTUCs are necessary.

## Conclusions

Contralateral metachronous UTUC after RNU is relatively rare. Our data suggest a potential role of DNA methylation in predicting contralateral UTUC recurrence after RNU. Unmethylated RASSF1A, no bladder recurrence prior to contralateral UTUC, history of renal transplantation, and preoperative renal insufficiency are independent risk factors of contralateral UTUC recurrence after RNU. For patients with such factors, follow up should be arranged closely, and examination of developing contralateral UTUC is necessary. Prophylactic contralateral nephroureterectomy is a reasonable choice for UTUC patients with renal transplant or end-stage renal failure.

## Consent

Written informed consent was obtained from the patient for the publication of this report and any accompanying images.

## Abbreviations

UTUC: Upper tract urothelial carcinoma
RNU: Radical nephroureterectomy
MI: Methylation index
CpG: Cytosine-guanine dinucleotide
CT: Computed tomography
MRI: Magnetic resonance imaging
eGFR: Estimated glomerular filtration rate
FFPE: Formalin-fixed paraffin-embedded
